# Stability data of extemporaneous oral suspension of pentobarbital in Syrspend SF Alka for imaging sedation procedure

**DOI:** 10.1016/j.dib.2021.106884

**Published:** 2021-02-17

**Authors:** Benjamin Querin, Arnaud Schweitzer-Chaput, Chahrazed Nedjar, Joël Schlatter

**Affiliations:** a Hôpital universitaire Necker – Enfants Malades, Assistance Publique des Hôpitaux de Paris (AP-HP), 149 rue de Sèvres, Paris, France; b Hôpital Paul-Doumer, Assistance Publique des Hôpitaux de Paris (AP-HP), 1 rue de l'hôpital, Liancourt, France

**Keywords:** Pentobarbital sodium, Pediatric, Sedation, Imaging, Stability, Oral suspension

## Abstract

Pentobarbital is used as an alternative in pediatric sedative imaging procedures. Pentobarbital is only available as pharmaceutical powder. Regardless of its clinical use, its manipulation is necessary byhospital pharmacists that must prepare adapted dosage forms for pediatrics. The data presented in this article suggest that extemporaneous suspensions of sodium pentobarbital in oral liquid base are stable for at least 120 days.

## Specifications Table

**Table:** 

Subject	Pharmacology, Pharmaceutical Science, Pediatrics
Specific subject area	Pharmaceutical Science
Type of data	Table Figure Text
How data were acquired	High-Performance Liquid Chromatography (HPLC)
Data format	Raw Analyzed
Parameters for data collection	Data on pentobarbital sodium stability in suspension through 120 days from preparation after storage at 5 ± 3 °C and 25 ± 3 °C
Description of data collection	The suspensions were prepared from pharmaceutical sodium pentobarbital powder using commercial Syrspend SF Alka vehicle. The suspensions were stored at 5 ± 3 °C and 25 ± 3 °C for 120 days and at scheduled times the samples were collected and analysed by HPLC.
Data source location	Paris, France
Data accessibility	Analysed data with the article. Additional tables and figures with supplementary data. http://dx.doi.org/10.17632/2bh8fmt97y.2

## Value of the Data

• The data support evidence on the pentobarbital chemical stability when the drug powder is suspended in Syrspend SF Alka reconstituted vehicle and stored for 120 days at two temperatures.
• The data enables hospital pharmacists and clinicians to use pentobarbital in a form suitable for pediatric radiology that addresses the absence of commercially available forms.
• The data reported in this study constitute a basis for further investigations to develop new formulations dedicated for pediatric patients for whom an oral liquid preparation is more suitable.

## 1 Data Description

Pentobarbital is a psychoactive drug with short-acting sedative effects in adult and pediatric patients. However, it is no longer marketed in Europe and in the United States, only the intravenous formulation of pentobarbital is available. Pentobarbital is used orally or rectally as an alternative in pediatric sedative procedures such as computed tomography or magnetic resonance imaging in infants to control pain, anxiety, and mobility [1], [2], [3], [4], [5]. Some information on the drug stability has been reported referring to the degradation of the drug under acidic conditions [6], [7], [8]. With respect to the necessity to have a pH upper 8.0 to protect pentobarbital degradation, an oral liquid preparation with a basic pH consisted of producing a stable oral suspension in Syrspend SF Alka vehicle buffered at a pH >7. Analytical results showed that pentobarbital content of the suspension remained at least > 95% for 120 days under both conditions, suggesting minimal or no loss of drug due by degradation or absorption (Table 1). No detectable change in odor, taste, color, or visible microbial growth was observed in any of the samples. In the suspension stored at room temperature and refrigerated, the final pH values on day 120 changed statistically from the initial pH (Table 1). The pH value of the drug suspension was above 9 during the 120-day study period, as recommended in Borodkin et al. [9]. This stability study responded to the proposal by Schlatter et al. to formulate a pentobarbital flavoring and sweetening suspension at pH >9 [8].

**Table 1:** Stability and pH of pentobarbital sodium 25-mg/mL SyrSpend SF Alka suspension at 22–25 °C and 4–8 °C.

	Mean ± S.D. initial								
		Mean ± S.D.% initial concentration remaining							
Storage	concentration (mg/mL)	Day 7	Day 14	Day 21	Day 30	Day 45	Day 60	Day 90	Day 120
22–25 °C	25.18 ± 0.79	97.89 ± 0.48	100.72 ± 0.67	102.20 ± 0.92	100.93 ± 1.14	98.93 ± 1.01	101.62 ± 0.44	98.78 ± 1.74	98.70 ± 0.95
pH	9.92 ± 0.01								9.69 ± 0.01*
4–8 °C	25.30 ± 0.78	99.77 ± 0.83	99.42 ± 0.63	99.93 ± 0.98	102.57 ± 1.43	101.08 ± 0.91	101.02 ± 0.43	99.25 ± 1.39	99.50 ± 1.22
pH	9.92 ± 0.01								10.02 ± 0.01*

⁎ *p* < 0.0001 (*t*-test comparing the means of initial and final pH).

## 2 Experimental Design, Materials and Methods

### 2.1 Materials

Pharmaceutical sodium pentobarbital powder (Ph. Eur.) obtained from Inresa Pharma (Bartenheim, France, lot 10,026/1111B479). Syrspend SF Alka purchased from Fagron (Rotterdam, Netherlands, lot 14/01-B03–300,337). This vehicle is a pre-measured alkaline suspending base for reconstitution and packaged in a graduated container allowing for compounding process directly into the dispersing container. Syrspend SF Alka is free of sugars and preservatives. It consists of calcium carbonate, modified food starch, and sucralose. It has low osmolality (<50 mOsmol/kg) and is specifically designed for acid-labile drugs. Purified water obtained from Fresenius Kabi (Versylene, Sèvres, France). All other chemicals and solvents used in this study were analytical grade.

### 2.2 Suspension preparation

Oral suspension of sodium pentobarbital 25 mg/mL was prepared by exactly weighed 2500-mg active pharmaceutical sodium pentobarbital powder and transferred into a mortar to obtain a fine and homogenous powder. The powder was transferred to the original container of vehicle and mixed. The mixed powder was then reconstituted with purified water to the final volume of 100 mL. The suspension was thoroughly mixed to obtain a uniform suspension. The method for suspension preparation was based on the general protocol indicated by the vehicle manufacturer [10]. Three identical samples of the formulation were prepared in the original vehicle container with a safety cap and stored at room temperature (25 ± 3 °C) and at refrigerated temperature (5 ± 3 °C).

### 2.3 Stability study

Following resuspension by shaking, aliquots of suspension from each of the stored samples were extracted on days 0, 7, 14, 21, 30, 45, 60, 90, and 120. After shaking by hand to prevent foaming, 1 mL of collected sample was diluted in 250 mL of purified water and was mixed for 10 s using a vortex before being analysed by HPLC. This method was reproduced from the previous publication of Schlatter et al. [11]. Stability was defined as retention of at least 90% of the initial drug concentration. All experiments were performed in triplicate.

### 2.4 Stability-indicating HPLC method

**Fig. 1:**
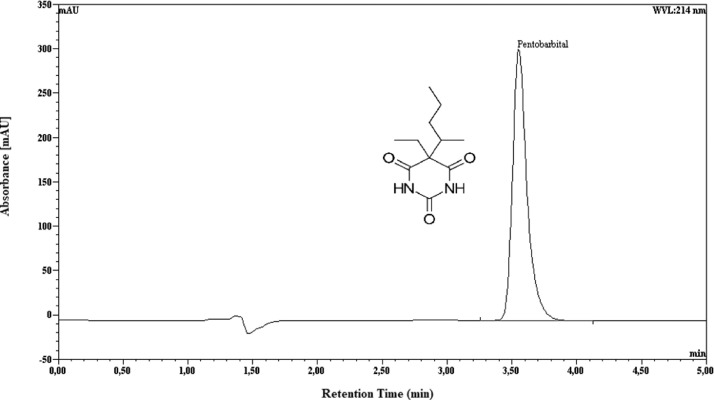
HPLC typical chromatogram of pentobarbital standard (25 mg/mL).

Pentobarbital concentration was measured using a previously published high-performance liquid chromatography (HPLC) stability-indicating method [12]. HPLC system (Dionex Ultimate 3000, Thermo Scientific, Villebon-sur-Yvette, France) was used with HPG-3200SD quaternary pump, WPS-3000TSL autosampler, and MWD-3000 variable wavelength detector. Data acquisition was carried out using in line Chromeleon® software (v6.80 SP2, Thermo Scientific). Chromatographic separation was achieved at 25 °C using a Nova-Pak (4 µm, 4.6 × 150 mm) C18 column (Waters, Guyancourt, France). The elution was run isocratically with a mobile phase consisting of 0.01 M potassium buffer pH 3 (400 mL) and methanol (600 mL) at a flow rate of 1.0 mL/min. The HPLC limit of detection was set at 214 nm. Chromatogram of pentobarbital in suspension is described in Fig. 1. Briefly, standard solution of pentobarbital sodium was prepared by accurately weighing 100 mg and diluted them with purified water to a final concentration of 0.1 mg/mL. Appropriate volumes of standard solution were diluted with purified water to yield 5 to 200 µg/mL for linearity. The injection volume was set at 25 µL.

### 2.5 Validation parameters of the HPLC method

**Fig. 2:**
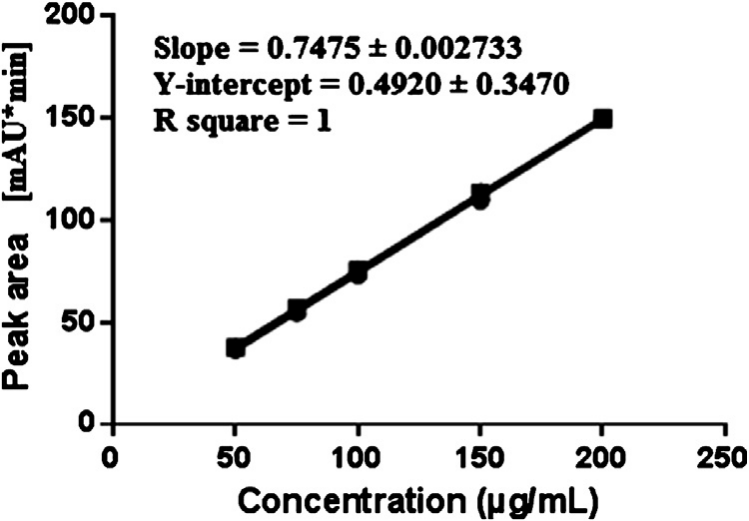
Pentobarbital calibration curve from 50 to 200 µg/mL.

**Fig. 3:**
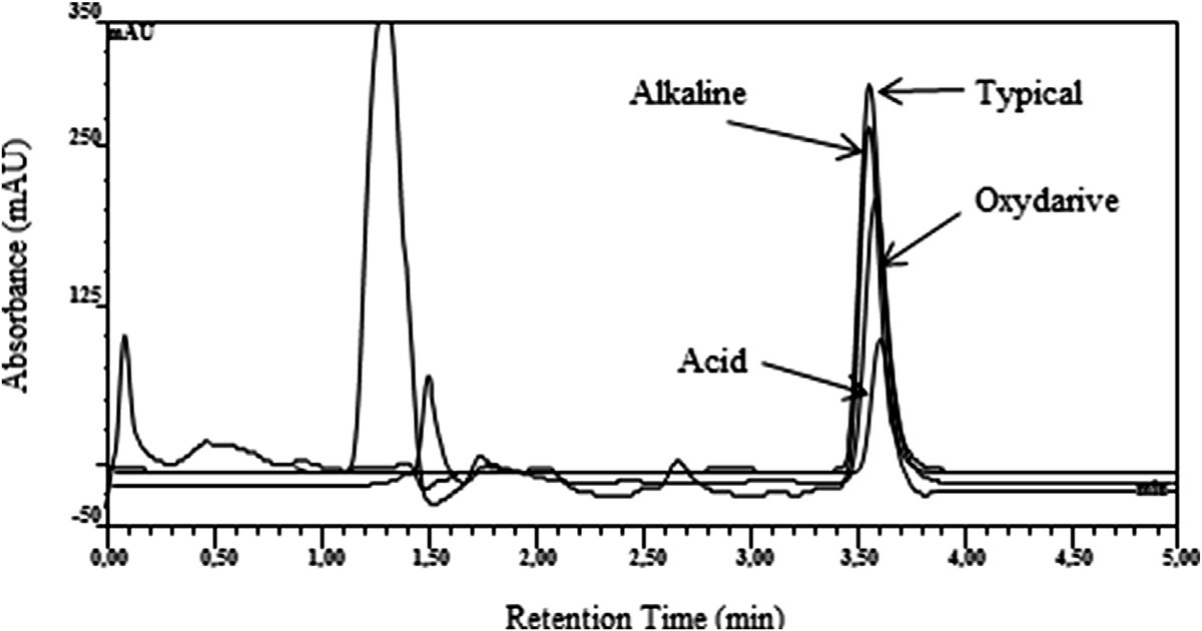
High-performance liquid chromatograms from forced degradation study.

The method was validated according to the International Council for harmonisation (ICH) Q2(R1) guidelines [13]. A stock solution of sodium pentobarbital (1 mg/mL) was prepared by dissolving the bulk drug in purified water. The linearity of the method was demonstrated using six different concentrations of pentobarbital sodium in a range between 50 and 200 µg/mL with R^2^>0.999 (Fig. 2, Table S3). Each concentration peak area was recorded in triplicate, and taken average area from triplicate injections. The lower limit of detection (LOD) and lower limit of quantification (LOQ) for pentobarbital was evaluated on standard deviation of the response and the slope of the calibration curve. LOD and LOQ were 1.5 mg/mL and 4.6 mg/mL, respectively. The percent relative standard deviation (%RSD) ranged from 1.2% to 2.5% at 50 µg/mL 0.9% to 1.1% at 150 µg/mL over the 3 separate days. The overall %RSD was less than 3% for all 3 days. To demonstrate the ability of the assay to be stability indicating, pentobarbital was subjected to acidic (12 N HCl), alkaline (10 N NaOH), and oxidative (3% H_2_O_2_) stress conditions for 48 h at 50 °C. Fig. 3 shows the chromatograms demonstrating the stability-indicating method. In these strong stress conditions, more than 50% loss of pentobarbital was observed in acidic and oxidative stress conditions, while loss due to alkaline stress condition was less than 10%.

### 2.6 Physical stability

The pH variationswere determined in the stability study on day 0 and day 120 with a calibrated pH meter (SevenExcellence digital pH meter, Mettler-Toledo, Viroflay, France). Samples were also observed for any visual change on each day analysis against black and white backgrounds. A statistical parametric *t*-test was used to compare the means of the pH using the software Prism 6 (Version 6.01, GraphPad Software, San Diego, USA).

## Ethics Statement

Not applicable.

## CRediT Author Statement

**Joël Schlatter:** Conceptualization, Methodology, Writing, Reviewing and Editing; **Benjamin Querin:** Data curation, Investigation; **Arnaud Schweitzer-Chaput:** Data curation, Formal analysis, Investigation; **Chahrazed Nedjar:** Software, Validation.

## Declaration of Competing Interest

The authors declare that they have no known competing financial interests or personal relationships which have, or could be perceived to have, influenced the work reported in this article.
